# Incidence and outcomes of neonatal group B streptococcal sepsis in Qatar-a multicentre study

**DOI:** 10.1186/s12887-025-05398-x

**Published:** 2025-01-17

**Authors:** Sajid Salim Thyvilayil, Anvar Paraparambil Vellamgot, Khalil Salameh, Sudheer Babu Kurunthattilthazhe, Abdurahiman Elikkottil, Liliana Llerena Dominguez, Dhanya Banarjee

**Affiliations:** 1 Department of Neonatology, Al Wakra Hospital, Hamad Medical Corporation, Doha, Qatar; 2 Department of Neonatology, Women’s Wellness and Research Centre, Hamad Medical Corporation, Doha, Qatar; 3 Department of Neonatology, Al Khor Hospital, Hamad Medical Corporation, Doha, Qatar; 4 Department of Neonatology, The Cuban Hospital, Hamad Medical Corporation, Doha, Qatar

**Keywords:** Group B streptococcus, Newborn, Early-onset sepsis, Late-onset sepsis, Incidence, Qatar, Neurodisability

## Abstract

**Background:**

Group B Streptococcus (GBS) is the most common cause of neonatal early onset sepsis in term infants and a major cause of late onset sepsis in both term and preterm infants.

**Aim:**

To estimate the incidence of GBSS among neonates born in Qatar between July 2015 and June 2020 (5 years). A secondary aim was to describe the outcomes of the affected babies.

**Materials and Methods:**

A retrospective chart review of all neonates born during the study period was performed with the help of medical records departments of the four main maternity hospitals in Qatar, where > 90% of the births occurred.

**Results:**

From 123,878 live births, 113 babies grew GBS in blood culture, during the first 90 days. 72 cases of early-onset GBS sepsis (EOGBSS) and 41 cases of late-onset GBS sepsis (LOGBSS) were identified. The estimated incidence of EOGBSS and LOGBSS were 0.58/1000 live births (95% CI 0.46- 0.73) and 0.33/1000 live births (95% CI 0.24- 0.45) respectively. The overall mortality was 7%, and the chart review identified severe neurodisability among at least 11% of survivors.

**Conclusion:**

The incidences of EOGBSS and LOGBSS in Qatar are 0.58/1000 live births (LB) and 0.33/1000 LB, respectively. The relatively high incidence of EOGBSS probably reflects the high rate of carrier state among pregnant mothers. We did not observe any significant change in incidence after introducing the universal maternal screening for GBS. The overall mortality was similar to previously published data. Further prospective studies are recommended.

## Introduction

Group B streptococcus (GBS) or Streptococcus agalactiae is a commensal, gram-positive bacterium commonly found in the genitourinary and gastrointestinal tracts of pregnant women [1, 2]. The role of GBS in neonatal sepsis was reported as early as the 1960s [3] and was recognized as a major pathogen by the 1970s [4]. Based on the time of onset of symptoms and possible mechanism of transmission, two main clinical variants are observed [5, 6]. Early-onset GBS sepsis (EOGBSS) occurs within the first six days of life, while late-onset GBS sepsis (LOGBSS) manifests between the 7th and 89th days of postnatal age. In addition, a late LOGBSS, which occurs beyond 90 days of age, has also been described.

EOGBSS is acquired through vertical transmission from the colonized mother. The rate of GBS colonization varies widely between countries and races. Around 12% of pregnant women in Southeast Asia are colonized, whereas the prevalence is as high as 35% in the Caribbean [2]. According to the Centers for Disease Control and Prevention, in the United States, 20–30% of pregnant women are colonized, around 50% of their babies acquire the bacteria from the mother, and 1–2% of these babies develop EOGBSS [5]. In addition to GBS colonization, several other risk factors have been linked to EOGBSS, including prematurity (< 37 weeks gestation), premature rupture of membranes (> 18 h) before delivery, chorioamnionitis, and the lack of appropriate intrapartum antibiotic treatment [7].

The introduction of intrapartum antibiotic prophylaxis (IAP) has resulted in a significant reduction in neonatal EOGBSS. In the United States, the use of IAP coincided with a decline in reported EOGBSS rates, from 0.7/1000 live births (LB) in 1997 [8] to 0.2/1000 LB in 2020 [9]. A similar reduction has been reported from around the world [10–12]. Compared to the risk-based approach for maternal IAP, universal screening of mothers is significantly more effective in preventing EOGBSS [13, 14]. In a systematic review of 90 studies on neonatal GBS incidence, Madrid et al. (2017) [15] estimated the worldwide incidence of EOGBSS and LOGBSS as 0.41/1000 LB and 0.26/1000 LB, respectively.

In more than 90% of babies with EOGBSS, clinical signs are apparent within the first 24 h of life and the manifestations include generalized sepsis, meningitis, and pneumonia [16]. LOGBSS may follow vertical or horizontal transmission. Compared to EOGBSS, additional organ involvement and meningitis are more frequent [17]. IAP is ineffective in preventing LOGBSS [18].

EOGBSS is associated with a 2–3% mortality rate among term babies and 20–30% among preterm babies [16, 19]. LOGBSS results in a 1–3% mortality rate among term babies and 5–8% among preterm babies [16, 19, 20]. In addition, survivors of GBSS are at high risk for adverse neurodevelopmental outcomes [19, 20].

Following the CDC recommendation in 2002 [21], Qatar implemented universal screening policy between 2003 and 2006. After this period, the country followed the risk-based approach recommended by the Royal College of Obstetricians and Gynecologists (RCOG) in 2003 [22]. In 2018, Qatar reinstated the universal screening policy. The estimated rate of GBS colonization in Qatar during 2008 was 34% [23]. However, this number is likely to vary over time, subject to changes in the proportion of the expatriate population.

The reported incidence of EOGBSS in the Middle East varies from 0.33 to 1.4/1000 LB. In a study comparing the incidence of EOGBSS between a risk-based and universal screening strategy, Abdelmaaboud et al. (2011) [24] estimated the incidence of EOGBSS in Qatar between 2003 and 2009 to be 0.51/1000LB.

In a multi-center, hospital-based prospective observational study, Hammoud et al. (2017) [25] estimated the incidence of EOGBSS as 0.7, 0.6, and 1.4 per 1000 LB in Saudi Arabia, United Arab Emirates, and Kuwait, respectively, with an overall rate of 0.9 per 1000 live births. In another study from Oman, Masroori et al. (2019) [26] observed the incidence of EOGBSS and LOGBSS to be 0.33 and 0.12/1000LB, respectively. Finally, in a retrospective review of 108,609 live births in Saudi Arabia, Al Luhidan et al. (2019) [27] estimated the overall incidence of GBSS as 0.51 / 1000 LB.

## Objectives

### Primary objective

1. To estimate the incidence and trend of early and late-onset neonatal GBS sepsis among neonates born in Qatar between July 2015 and June 2020 (5 years)

### Secondary objectives

1. To describe the clinical characteristics and outcome of infants with GBS sepsis.
2. To estimate the predictors of mortality in Neonatal GBS sepsis.

## Methods

### Study design

This was a retrospective medical records review of all consecutive live-born babies born in four Hamad Medical Corporation (HMC) hospitals in Qatar between July 2015 and June 2020.

### Study setting

HMC is the chief healthcare provider in Qatar and serves a population of approximately 2.7million. During the study period, more than 90% of childbirths in Qatar occurred in four HMC facilities, namely Women's Wellness and Research Centre (WWRC), Al Wakra Hospital (AWH), Al Khor Hospital, and The Cuban Hospital (TCH). Since 2014, the healthcare system in Qatar, including hospitals, primary health centers, and emergency centers, has been connected through the electronic Clinical Information System, Cerner Millennium.

The study was conducted in the above four HMC hospitals with maternity facilities. After obtaining HMC Institutional Review Board (IRB) approval, each facility's Medical Records Department (MRD) was approached to obtain the number of live births and the health card numbers of all live-born babies between July 2015 and June 2020. The data section of each MRD screened every electronic file for any positive blood culture during the first 90 days of life. From this data, babies with GBS bacteremia were selected manually.

Since the search was exhaustive, involving 123,878 files, only positive blood culture results were included for screening. Positive cerebrospinal fluid culture growth without bacteremia was not included in this screening.

Further details were collected by reviewing the electronic medical records of each case.

As per the HMC clinical guidelines for neonatal sepsis, blood cultures were performed based on clinical features or in the presence of significant perinatal risk factors. Lumbar puncture (LP) was recommended for all blood culture-positive cases and cases of late-onset sepsis. The HMC laboratories used standard technologies for bacterial isolation, identification, and confirmation, including the BD Phoenix system and MALDI-TOF MS (Matrix Assisted Laser Desorption Ionization Time Flight Mass Spectrometry) by Bruker, USA.

### Consent

The design was retrospective, and all the subjects were de-identified by coding. Hence, the IRB of HMC, Qatar, exempted the study from informed consent.

### Inclusion and exclusion criteria

Live-born babies born in the above four HMC facilities between July 2015 and June 2020 were included in the study.

Stillbirths, abortions, and babies born outside the four facilities were excluded.

### Variables

Each case and the corresponding maternal files were reviewed.

A. Maternal data

The maternal variables collected included age at the time of delivery, gestational age, parity, chorioamnionitis, total duration of premature rupture of membranes if any, GBS carrier status (categorized as positive, negative, and unknown), timing and type of intrapartum antibiotics if any, peak intrapartum temperature, and mode of delivery. GBS status was considered positive if urine or vaginal swab was positive during the current pregnancy. If a low vaginal swab was done within five weeks of delivery and was negative, it was categorized as negative. All others were considered to have an "unknown" status.

B. Newborn data.

The neonatal data collected includes: date of birth; gestational age; birth weight; sex; nationality; Apgar score at birth; any need for positive pressure ventilation at birth; age upon first clinical presentation (in hours for EOGBSS and in days for LOGBSS); the presenting symptoms (respiratory distress, fever, lethargy, poor sucking, seizures, and other symptoms as specified); age upon first positive blood culture (in hours for EOGBSS and in days for LOGBSS); age upon second positive blood culture if any; time to positive gram stain reports (from blood collection to the first report of gram stain result in the system); total white blood cell (WBC) count within the first 24 h of presentation; any repeat WBC after 24 h; peak C-reactive protein (CRP) within 48 h of clinical presentation; lowest recorded platelet count; any increase in CRP recorded over a 48-h period; cerebrospinal fluid (CSF) study (CSF was considered abnormal if positive culture or latex agglutination or evidence of meningitis by cell count/biochemistry in the absence of a blood-stained sample); type and duration of antibiotics; brain imaging – timing and abnormality detected; total duration of respiratory support; duration of mechanical ventilation; need for a central line, inotropes, and blood transfusion; need for intensive care; length of stay; and mortality.

We reviewed all files to identify follow-up visits beyond 18 months of chronological age. In addition, for infants who had at least three visits with the pediatrician after 18 months of age, we examined the documentation for any evidence of major neurodisabilty, defined as cerebral palsy, significant hearing or a visual impairment, or intellectual disability, as documented in the file.

## Variable definitions

- *Early-onset GBS sepsis (EOGBSS)*: Neonate with positive blood culture from birth to 6 days of age. Isolated CSF cultures were not included in this study.
- *Late-onset GBS sepsis (LOGBSS)*: Neonate who becomes culture positive from 7 to 89 days of age. Isolated CSF cultures were not included in this study.
- *Pneumonia*: if documented as pneumonia in the final diagnosis, irrespective of the radiologist’s report.
- *Confirmed meningitis*: GBS isolated in culture or PCR or latex agglutination or abnormal CSF in the presence of GBS bacteremia.
- *Probable meningitis*: if documented as meningitis in the final diagnosis, irrespective of whether a CSF study was performed.
- *Neurodisability*: cerebral palsy, significant hearing or a visual impairment, or intellectual disability, as documented in the file, among babies who had at least three documented follow-ups with a pediatrician after 18 months of chronological age.
- *Fever*: Axillary temperature ≥ 38^0^C.
- *Leukopenia*: White Blood Cell Count < 5 × 10 ^3^/L.
- *Neutropenia*: Absolute Neutrophil Count < 1 × 10 ^3^/L.
- *Thrombocytopenia*: Platelet count < 150 × 10 ^3^/L.

### Bias

Bias related to the retrospective design is acknowledged. Single-center or lab-based studies are likely to result in selection bias, leading to an underestimation of the true population incidence. Our study involved four centers with different levels of care, and all live-born babies during the study period were screened. The results are more likely to be representative of the true population incidences. However, we did not include cases with isolated positive cerebrospinal fluid cultures. Although this is unlikely to affect the EOGBSS estimation, a slight underestimation of the LOGBSS is expected.

### Sample size estimation

Based on a previously published study, the EOGBSS in Qatar was 0.51/1000 LB. Since our secondary objective was to describe the outcome of GBSS, we included a 5-year data period with approximately 126,000 LB, expecting 75 to 100 cases of GBSS.

### Statistical analysis

Statistical analysis was performed using the statistical package SPSS 22.0 (SPSS Inc., Chicago, IL). Anonymous data were collected and entered into a standardized excel data collection chart, which was designed based on the study design and objectives. Since the study was descriptive, no formal hypothesis was tested. The incidence of GBSS for every 1000 live births with a 95% confidence interval was estimated using Wilson's score interval. Descriptive statistics were used to summarize all demographic data, risk factors, and outcome data. The results were reported as mean and standard deviation (SD), median (IQR) or frequencies and percentages depending on the type of data. The association between two or more categorical variables was assessed using the chi-square (× 2) test or Fisher's exact test as appropriate. An unpaired t-test was used to compare continuous variables related to EOGBSS and LOGBSS. Data presented as median (IQR) were tested using the Mann–Whitney U test. Multiple logistic regression was used to assess the predictors of mortality. All presented *P* values were two-tailed, and *P* values less than 0.05 were considered statistically significant.

#### Missing data

Patients with missing variables were excluded from the study.

#### Consent and ethical considerations

This was a retrospective chart review. All the subjects were de-identified by coding. Hence the Institutional Review Board (IRB) of Hamad Medical Corporation waived off the need for consent from the subjects.

## Results

123,878 live births were recorded during the study period. Among them, 938 had positive blood cultures within the first 90 days of life. This included some babies who had positive culture on more than one occasion and babies with non-significant organisms. The overall EOS incidence was not estimated in this study. Among 188 babies with positive blood cultures on or before six days of life, 72 babies grew GBS. Among all the babies with positive cultures between 7 and 89 days, 41 grew GBS.

The overall incidence of early and late-onset GBSS were 0.58/1000 live births (95% CI 0.46, 0.73) and 0.33/1000 live births ( 95% CI 0.24,0.45), respectively (Table 1).

**Table 1:** Incidence of early and late onset GBSS

Year	Total live births	EOGBSS	LOGBSS	Total GBSS cases	EOGBSS /1000 live births (95% CI)	LOGBSS/1000 live births (95% CI)	Total GBSS /1000 live birth (95% CI)
July-Dec 2015	11,596	13	7	20	1.1 (0.65, 1.92)	0.6 (0.29, 1.24)	1.7 (1.1, 2.7)
2016	23,954	11	12	23	0.46(0.26,0.82)	0.51 (0.29, 0.87)	0.97 (0.64, 1.4)
2017	25,295	11	7	18	0.43(0.24,0.78)	0.28 (0.13, 0.57)	0.71 (0.45, 1.1)
2018	24,725	17	7	24	0.69 (0.43,1.1)	0.28 (0.13, 0.58)	0.97 (0.65, 1.4)
2019	25,744	16	5	21	0.62 (0.38, 1)	0.19 (0.08, 0.45)	0.82 (0.53, 1.2)
Jan -June 2020	12,564	4	3	7	0.32(0.12,0.82)	0.24(0.08, 0.7)	0.55(0.27, 1.1)
Total	123,878	72	41	113	0.58(0.46,0.73)	0.33 (0.24, 0.45)	0.91 (0.75, 1.09)

*GBS* Group B streptococcus, *EOGBSS* Early-onset GBS sepsis, *LOGBSS* Late-Onset GBS Sepsis, *CI* Confidence interval

There was no significant reduction in the number of EOGBSS cases after adopting the universal GBS screening of pregnant mothers (Table 2).

**Table 2:** Comparison of EOGBSS incidence before and after implementation of universal GBS screening

Period	Live births	EOGBSS	Incidence of EOGBSS	*P* value
Before implementation (2015–2017)	60,845	35	0.57/1000 LB	0.73
After implementation (2019–2020)	38,308	20	0.52/1000 LB	

*EOGBSS* Early-Onset GBS sepsis

**Fig. 1:**
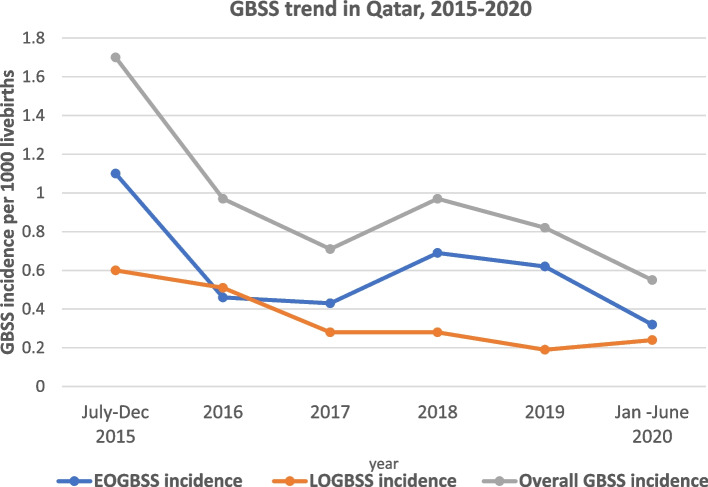
GBSS trend in Qatar, 2015–2020

The reported incidences of EOGBSS and LOBSS were high in 2015. After 2015, no noticeable trend was observed (Fig. 1).

35% of mothers were of Qatari nationality. The mean maternal age was 27 ± 5 years. 55.8% were nulliparous, and 92.9% had regular antenatal visits. Among 44 mothers with known GBS status, only 43% were GBS carriers. 55.8% of babies were born by Cesarean delivery, and 50.4% were males. The mean birth weight was 2.62 ± 0.89 kg (Table 3).

**Table 3:** Baseline maternal and neonatal characteristics

Variables	N = 113
Qatari national, No (%)	40(35)
Maternal age, mean (± SD)	27.5(5)
Gestational age, Mean (± SD)	36.16(4.5)
Prematurity, No (%)	36 (31.9)
Nulliparity, No (%)	63(55.8)
regular antenatal visits, No (%)	105(92.9)
GBS status known, No (%)	44 (38.9)
GBS positive, No (%)	19/44 (43.2)
Cesarean delivery, No (%)	63(55.8)
Instrumental vaginal delivery, No (%)	13(11.5)
Male sex, No (%)	57(50.4)
Birthweight, Mean (± SD)	2.62(0.89)

*GBS* Group B Streptococcus

Overall, 11.5% of the mothers in the study experienced intrapartum fever. Spontaneous rupture of membranes (SROM) was noted in 47.8% of the cases, and clinical chorioamnionitis was observed in 11.5%. The GBS status was known for only 39% of all mothers, and out of those tested for GBS, 44% were positive. There was no significant difference in the maternal GBS carrier rate between the EOGBSS and LOGBSS groups (*p* = 0.56). Additionally, 31.9% of babies in the study were premature. For obvious clinical reasons, SROM (*p* = 0.01) and chorioamnionitis (*p* = 0.023) were more frequent in the EOGBSS group, while prematurity (*p* = 0.017) and very low birth weight (*p* = 0.01) were significantly higher in the LOGBSS group (see Table 4 for details).

**Table 4:** Sepsis risk factors – Comparison between EOGBSS and LOGBSS

Variable	**All GBSS** **N = 113**	**Comparison EOGBSS vs. LOGBSS**			
		**EOGBSS** **N = 72**	**LOGBSS** **N = 41**	***p*** **-value**	**OR (95%CI)**
Intrapartum fever (38 or more), N (%)	13 (11.5)	11 (15.3)	1(2.4)	0.05	7.2 (0.9,58)
SROM, N (%)	54(47.8)	41(56.9)	13(31.7)	0.01	2.85 (1.27, 6.38)
ROM > 18 h, N (%)	19 (16.8)	14 (19.2)	5(12)	0.32	1.74(0.57,5.2)
Labor induction, N (%)	24 (21.2)	16 (22)	8(19.5)	0.73	1.18 (0.58,3)
GBS positive among all tested, N (%)	19/44 (44%)	11/22 (57.9)	8/22(34.2)	0.37	1.75 (0.5,5.8)
Did not receive adequate intrapartum antibiotics; among all indicated cases, No (%)	24/46 (52)	17/30 (56)	8/16 (50)	0.56	1.4 (0.45, 4.8)
Clinical chorioamnionitis, N (%)	13 (11.5)	12 (16.7)	1(2.4)	0.023	8(1,63)
VLBW, N (%)	18 (15.9)	7(9.7)	11 (26.8)	0.01	0.29 (0.1,0.8)
Prematurity, N (%)	36 (31.9)	20(27.6)	16(39)	0.017	0.29 (0.1 to 0.83)

*SROM* Spontaneous Rupture of Membranes, *ROM* Rupture of Membranes, *VLBW* Very Low Birth Weight *OR* Odds Ratio, *CI* Confidence interval

Clinical and laboratory characteristics are summarised in Table 5. Although 21% of babies required positive pressure ventilation (PPV) at birth, only one baby had a 5-min Apgar score < 5, and there was no significant difference in the need for PPV between EOGBSS and LOGBSS cases. The median age at clinical presentation was 2.5 h (IQR 0, 15) for EOGBSS and 21 days (IQR 15, 51) for LOGBSS. Nearly half of the EOGBSS cases were symptomatic at birth, and 90% presented within 48 h of birth. Among LOGBSS cases, 50% presented by 30 days of age and 85% by 60 days. Eighty percentage of LOGBSS and 6.9% of EOGBSS cases were already discharged home before they presented with sepsis. Respiratory distress was the most frequent presenting symptom of EOGBSS. 82% of EOGBSS and 45% of LOGBSS cases presented with respiratory distress (P < 0.001). Fever (temperature ≥ 38 °C) was much more frequent among LOGBSS cases (P < 0.001). Pneumonia was significantly more common in EOGBSS cases (P < 0.001). Lumbar puncture was performed for 97 babies (85.8%). For 16 babies, LP was not done due to parental refusal, critical general condition, or the treating physician's discretion. Twenty-six babies were confirmed to have meningitis, and an additional 22 babies were diagnosed with probable meningitis. The proportion of confirmed meningitis was higher in EOGBSS, but the difference did not reach statistical significance (*p* = 0.054).

**Table 5:** Clinical presentation and laboratory parameters

Clinical variable		All GBSS (N = 113)	Comparison-EOGBSS vs LOGBSS			
			**EOGBSS (N = 72)**	**LOGBSS (N = 41)**	***p***	**OR (95%CI)**
1 min Apgar < 5, No (%)		12(10)	9(12.5)	3(7.3)	0.53	1.8(0.46–7)
5-min Apgar score < 5, No (%)		1(0.8)	1(1.4)	0(0)	1	
Any PPV at birth, No (%)		24(21)	18(25%)	6(14.6)	0.19	1.94(0.7 -5.4)
Age upon clinical presentation, median (IQR)			2.5 h (0–15)	21 days (15–51)		
Age of presentation, Cumulative No (%)	0 h		35(49)			
	1–12 h		51(70)			
	13- 24 h		62(86)			
	25–48 h		65(90)			
	49 h -6 days		72(100)			
	7–14 days			8(19.5)		
	15–30 days			24(51)		
	31 -60 days			35 (85)		
	61–90 days			41 (100)		
Presented after initial post-delivery discharge		38(33.6)	5 (6.9)	33 (80)		
Symptoms upon first clinical suspicion, No (%)	Fever 38-degree Celsius or more within 12 h of presentation	51 (45.1)	16(22.2)	35 (85.4)	< 0.001	0.05 (0.02 -0.13)
	Respiratory distress	78(69)	59(81.9)	19(46.3)	< 0.001	5.3(2.2–12.4)
	hypo-activity/poor sucking	65(57.5)	33 (48.8)	32 (78)	< 0.001	0.24 (0.09—0.57)
	Irritability	14(12.4)	2(2.8)	12(29.8)	< 0.001	0.07 (0.02 to 0.23
	Apnoea/bradycardia/desaturation	9(8)	3(4.2)	6(14.6)	0.07	0.24 (0.06 to 1.02)
	Seizures (within 72 h)	22(19.5)	16(22.2)	6(14.6)	0.33	1.67 (0.59–4.66)
	soft tissue or urinary tract infections (No.)	6 (5.3)	0	6 (14)	0.03	0.35 (0.25–0.45)
	Asymptomatic (No.)	4	4	0	0.29	
Pneumonia, No (%)		43(38)	37(52)	6(14)	< 0.001	6.35 (2.37–16.9)
Meningitis (confirmed) No (%)		27 (23.9)	13(18)	14(34)	0.054	0.43 (0.17–0.91)
Meningitis (confirmed + probable), No (%)		48 (42)	28(38)	20(48)	0.306	0.69 (0.3,1.45)
Metabolic acidosis N (%)		54/85 (63)	40/57(70.7)	14/28(50)	0.06	2.35 (0.93–5.98)
Thrombocytopenia (< 150 × 10 ^3^/L), N (%)		24(22.5)	22(25%)	7(17.5)	0.37	1.57 (0.59–4.2)
Neutropenia (< 1 × 10 ^3^/L), N (%)		13(11.9)	7(10.1)	6(15)	0.45	0.64 (0.19–2)
Leukopenia (< 5 × 10 ^3^/L), N (%)		28(25.5)	23(32.9)	5(12.5)	0.02	3.4 (1.19–9.2)
Peak CRP (in mg/dL), mean (SD)		61(57)	62(56)	58(58)	0.79	Mean difference 3.2 (-21,28)

*EOGBSS* Early Onset GBS sepsis, *LOGBSS* Late Onset GBS Sepsis, *PPV* Positive pressure Ventilation, *IQR* Interquartile range, *CI* Confidence Interval

Occurrences of metabolic acidosis, thrombocytopenia, neutropenia, and peak CRP were comparable between early and late onset GBS sepsis. Leukopenia was more common in EOGBSS (32.9% vs 12.5%, *p* = 0.02).

Table 6 summarises the outcomes of affected babies. The need for intensive care admission (*P* < 0.001) and respiratory support (*P* < 0.01) was higher for EOGBSS. There was no difference in the need for central lines (*P* = 0.21), inotropes (*P* = 0.52), blood products (*P* = 0.26), or fluid boluses (*P* = 0.05). The median length of stay was comparable (15.5 days in EOGBSS vs. 20 days in LOGBSS, *P* = 0.24).

**Table 6:** Short and long-term outcomes of babies with GBSS

Clinical variable	All GBS (N = 113)	Comparison-EOGBSS vs LOGBSS			
		EOGBSS N = 72	LOGBSS N = 42	*P* value	95% CI
Intensive care admission, No (%)	95(84)	71 (98.6)	24(58.4)	< 0.001	50 (6.35–398)
Any respiratory support, No (%)	75 (66)	60 (83)	15(36)	< 0.001	8.67 (3.56–21)
Mechanical ventilation (MV), No (%)	35(23.9)	26(36.1)	9(22)	0.11	2(0.8—4.3)
Duration of MV in hours, Median (IQR)	60(14,120)	46(12,120)	96(54,288)	0.22	
Total duration of respiratory support in days, Median (IQR)	4(1.9,15)	6.5(2,22.5)	7(5.5,55.5)	< 0.01	
Central line, No (%)	36(31.2)	20 (27.8)	16(39.9)	0.21	0.6 (0.26–1.34)
Inotrope use No (%)	17(15%)	12(16.7)	5 (12.2)	0.52	1.44 (0.47–4.4)
Blood products, No (%)	8 (7.1)	7 (9.7)	1 (2.4)	0.26	4.3(0.5–35)
Fluid bolus, No (%)	23(20.4)	19 (26.4)	4(9.8)	0.05	3.36(1.05–10.5)
Antibiotic duration in days, Median (IQR)	14 (10.21)	14(9.25,21)	21(14,22.5)	0.14	
Length of stay in days, Median (IQR)	19(2,23)	15.5(11–21)	20(13.5–2.8)	0.24	
Proportion of babies with documented follow-up till18 months or beyond, No (%)	98(86.2)	61 (84.7)	37 (90.2)	0.41	0.62(0.18 -2)
Age in years at the time of data collection, mean (± SD)	4.9(1.4)	4.2(1.3)	5.4(0.77)	0.19	
Death, No (%)	8(7.1)	7 (9.7)	1(2.4)	0.26	4.3 (0.51–36.3)
Cerebral palsy and/or major cognitive defect, No (%)	11(11.2)	6 (9.8)	5(13.5)	0.58	0.69(0.19–2.4)
Combined death/major neuro-disability, No (%)	19(18.1)	13(19)	6(15.8)	0.64	(0.44–3.7)

*EOGBSS* Early Onset GBS sepsis, *LOGBSS* Late Onset GBS Sepsis, *MV* Mechanical Ventilation, *IQR* Interquartile range, *CI* Confidence Interval

Seven babies (10%) with EOGBSS and one baby (2.4%) with LOGBSS expired during the initial admission for sepsis (P 0.26). Five babies were born premature. No deaths were recorded after the initial discharge.

Electronic medical records were reviewed to assess the availability of follow-up data beyond 18 months of chronological age. At least 3 documented pediatric clinic follow-ups after 18 months of age were available for 86% of EOGBSS and 90% of LOGBSS cases. Physician notes were reviewed to identify any documented major neurologic disabilities, such as cerebral palsy or major cognitive or sensory defects. The mean age at the review time was 4.2 years for EOGBSS and 5.4 years for LOGBSS. 11 babies (11%) had major neurodisability, which included ten babies with cerebral palsy with or without global delay and one late preterm baby with severe visual impairment and mild delay. Seven of the 11 babies (63%) were preterm, and 3 of the preterm babies were of < 30 weeks gestation. There was no statistically significant difference in the rate of major neurodisability between the two groups (9.8% for EOGBSS and 13.5% for LOGBSS, *P* = 0.58), or in the combined incidence of death and major neurodisability (19% for EOGBSS vs. 15.8% for LOGBSS, *P* = 0.64).

In bivariate analysis, lower birthweight, lower gestational age, need for fluid bolus, mechanical ventilation, high CRP, leukopenia below 5 × 10 ^3^/L, thrombocytopenia below 150 × 10 ^3^/L and meningitis were found to be significant predictors of combined mortality and neurodiability, whereas maternal age, chorioamnionitis, instrumental delivery, need for positive pressure ventilation at birth, gender and type of sepsis were not significant predictors. However, multiple logistic regression showed that only meningitis (adjusted OR 11.53, p = 0.01),need for fluid bolus (adjusted OR 16.8, P 0.01), and peak CRP (adjusted OR 1.01, *p* = 0.03) were significant predictors of combined death and neurodisability, when adjusted for birth weight, gestational age, gender, leukopenia, thrombocytopenia and need for positive pressure ventilation at birth (Table 7).

**Table 7:** Predictors of the combined outcome of mortality plus neurodisability in neonatal GBSS

Variable	*P* value	Adjusted OR	95% confidence interval
Gestational age in weeks	0.81	0.94	0.55–1.57
Type of sepsis—EOS/LOS	0.89	0.86	0.10–7.15
Gender	0.37	2.27	0.38–13.4
Birthweight in grams	0.95	0.93	0.07 -12.47
Positive pressure ventilation at birth	0.23	3.73	0.43–32.08
Highest CRP	0.03	1.01	1.00–1.03
Need for fluid bolus	0.01	16.80	1.98–142.34
Need for mechanical ventilation	0.17	3.63	0.57–23.11
Meningitis	0.01	11.53	1.63–81.61
Constant	0.56	0.02	

## Discussion

The early and late onset GBS incidences in Qatar are comparable to data from different international centres (Table 8). However, we could not find a plausible explanation for the high rate recorded during 2015. In a recently published study from Qatar, Ali et al. (2022) [28] reviewed 196 invasive blood stream infections involving all age groups. They observed an increasing trend from 1.48/100000 population in 2015 to 2.09/100000 population in 2018.

**Table 8:** GBSS incidence: Comparison of the current study to published data

Authors	Country	Study period	Study method	EOGBSS	LOGBSS	Comments
Present study	Qatar	2015–2020	Retrospective review	0.58	0.33	▪ All live-born babies during the study period were screened -Birth registry based
CDC 2020 [9]	USA	2020	Prospective surveillance	0.2	0.28	Definitions: ▪ EOGBSS – 6 days or less ▪ LOGBSS- 7 to 90 days
O’Sullivan et al.,2019 [29]	UK	2014–2015	Prospective surveillance	0.57	0.37	
Sing et al., 2019 [30]	Australia	2002–2012	Prospective Surveillance	0.43		▪ Definition EOGBSS: 48 h, ▪ data collected from centres in major cities, not representative of the more urban population ▪ There was no significant trend
Ding et al., 2023 [31]	China	2016–2022	Systematic review	0.28	0.13	▪ Included 1,314,216 live births in Mainland China
Sikias et al., 2022 [32]	France	2019–2021	Prospective observational study	0.16		▪ Only 34 weeks and more gestation included, ▪ EOGBSS definition: < 72 h
Abdelmaaboud et al., 2011 [24]	Qatar	2003–2009	Retrospective review	0.51		▪ EOGBSS definition—< 72 h ▪ Laboratory-based
Hammoud MS et al., 2017 [25]	Middle East (UAE, Kuwait, Saudi Arabia)	2013–2015	Multi centre, prospective, observational	UAE: 0.9, Kuwait:2.64 Saudi: 0.4		▪ EOGBSS < 72 h ▪ Only cases admitted to NICU were included
Manroori et al., 2019 [26]	Oman	2006–2016	Retrospective review	0.33	0.12	▪ EOGBSS- up to 6 days ▪ LOGBSS- 7 to 90 days ▪ Only NICU admissions were considered
Almudeer et al., 2020 [33]	Saudi Arabia	2011–2018	Retrospective review	0.74		▪ Overall GBSS, up to 28 days ▪ Only NICU admissions were considered

*CDC* Centers for Disease Control and Prevention, *US* United States, *UK* United Kingdom, *UAE* United Arab Emirates. *EOGBSS* Early-onset GBS sepsis, *LOGBSS* Late-Onset GBS Sepsis

The data also depends on the definition used and the study methodology. Studies with a definition of 72 h for EOGBSS would naturally miss cases presenting between the fourth and sixth day of life. Single-center-based, NICU-based, and lab-based studies are also likely to under-report the incidence. There was no significant decrease in EOGBSS cases after implementing universal maternal GBS screening in 2018 (0.57 vs. 0.52 per 1000 LB, p 0.73). The previous study in Qatar by Abdelmaaboud et al. (2011) [24] also did not observe a significant change in incidence of EOGBSS after shifting to the risk-based approach (2006–2009) from universal screening policy (2003–2005) (0.53 during universal screening vs. 0.57 during risk-based screening, p 0.28). Schrag et al. (2002) [13] and Hasperhoven et al. (2020) [14] have reported a significant reduction in EOGBSS cases after adopting the universal screening strategy. However, this policy is not universally accepted. The RCOG recommends the risk-based approach as a cost-effective strategy for preventing EOGBSS [22]. In short, the strategy should be driven by accurate data obtained through good-quality prospective research conducted in the country concerned.

The incidence of LOGBSS also did not vary significantly over the study period. Except for a relatively higher incidence during 2015, the LOGBSS rate remained between 0.5 and 0.33/1000LB during 2016–2020. The introduction of IAP has not affected the incidence of LOGBSS [16]. The rate of LOGBSS in Qatar is consistent with the incidence worldwide.

The mean maternal age was 27 ± 5 years. Maternal age below 18 years has been identified as a strong predictor of EOGBSS [34]. Thirty-five percent of mothers were of Qatari nationality, and the remaining patients belonged of 18 different nationalities. Ethnicity and culture are important determinants of GBS carrier state. Mothers of African origin have a significantly increased colonisation rate and EOGBSS [35].

Maternal carriage of GBS is a prerequisite for neonatal infection. Prematurity, prolonged rupture of membranes (> 18 h), chorioamnionitis, and intrapartum fever are well-known risk factors for EOGBSS [35]. In this study, 72 babies were diagnosed with EOGBSS. The observed risk factors included prematurity (27.6%), clinical chorioamnionitis (11.5%), intrapartum fever (11.5%), premature rupture of membranes > 18 h (16.8%), and GBS colonisation (58% of 22 mothers who were tested). In a population-based study involving a large cohort of 377 infants with EOGBS, Heath et al. (2004) [36] observed that 42% had no significant risk factors for EOGBSS. In our study, nearly half of the mothers who were screened for GBS were negative. In a systematic review, Valkenburg-van et al. (2010) [37] observed positive and negative predictive values of 43–100% and 80–100%, respectively, with higher accuracy when tested closer to delivery.

The pathogenesis and risk factors of LOGBSS are not well defined [17]. In our study, 34% of mothers who tested for GBS in the LOGBSS group were carriers, which was similar to those with EOGBSS. LOGBSS cases were more likely to be premature (39% vs. 27.6%, *p* 0.017). Spontaneous rupture of membranes (SROM) and chorioamnionitis were less frequent in LOGBSS. Maternal GBS carrier state increases the risk of neonatal carriage and, thus LOGBSS [17]. Pintye et al. (2016) [38] reported that 40–50% of all LOGBSS occur among premature neonates. Berardi et al. (2016) [[3) [17] also suggested cross-infection from mothers as an important source of LOGBSS, with a possibility of transmission through breast milk.

We observed that nearly half of the EOGBSS cases were symptomatic at birth, with 86% presenting within 24 h and 90% within 48 h of birth. Only 10% presented between 48 h and six days. Among the seven babies who presented after 48 h, five had already been discharged home before the onset of symptoms. Heath et al. (2004) [36] reported that half of EOGBSS cases were symptomatic at birth, and 98% presented within 12 h of age. In the multi state surveillance from the US, Nanduri et al. (2019) [16] reported that 94.8% were diagnosed within 48 h of life. In our study, 5 of the seven babies who developed symptoms after 48 h were already discharged home before they presented. The overall outcome of this small group was not different from the rest of the cohort. In the same multi centre surveillance [16], 0.3/100000 live births were discharged home before presenting with EOGBSS.

Respiratory distress (81.9%) and hypo-activity (48.8%) were the most common presenting symptoms in EOGBSS. Fever was present in 22%. Pneumonia and confirmed meningitis were diagnosed in 52% and 18% of cases, respectively. Four babies (5.5%) remained asymptomatic, and a blood culture was sent due to significant risk factors for EOGBSS. In contrast, Nanduri et al. (2019) [16] observed bacteremia without focus as the presenting type in 83% of EOGBSS and meningitis was observed in 9.5%. We included all documented cases of pneumonia, irrespective of the radiology report. Pneumonia was suspected in any case of EOGBSS requiring respiratory support with suspicious x-ray findings in bedside review. The rate of meningitis is comparable to that reported by Nanduri et al. (2019) [16]. Madrid et al. (2017) [15] observed meningitis among 16% of babies with EOGBSS.

LOGBSS cases presented at a median age of 21 days (IQR 15–51 days). Fever (85.4%) and hypo-activity (78%) were the most common presenting symptoms. Eight premature infants, who were still not discharged home after post-delivery hospital admission, presented with apnea, bradycardia, and desaturation as the predominant symptoms. The proportion of confirmed meningitis was higher in LOGBSS (34% vs. 18%), but the difference did not reach statistical significance (p 0.054). Overall, meningitis was diagnosed in 48% of all LOGBSS cases. Two cases of LOGBSS had myositis of the thigh; one had orbital cellulitis, and another three babies presented with associated urinary tract infection. Nanduri et al. (2019) [16] reported a median presenting age of 34 days (IQR 20–49 days), and bacteremia without focus was the most common presentation. GBS was isolated from CSF in 20.7%, meningitis was diagnosed in 31.4% of cases, soft tissue infection was reported in 1.8% and 7% of babies had meningitis without bacteremia [16]. Our study included only cases with bacteremia, and we estimate that 2–3 cases of LOGBSS meningitis without bacteremia might have been missed during the 5-year study period.

In a prospective cohort study involving 311,893 births, Berardi et al. (2013) [17] described 100 neonates with LOGBSS. Of them, 57% had bacteremia without focus, 34% had meningitis, and 7% had soft tissue infections. In the systematic review involving 6900 cases of neonatal GBSS, Mardrid et al. (2017) [15] observed meningitis among 43% of LOGBSS cases.

Abnormal laboratory markers, including increased CRP, thrombocytopenia, and metabolic acidosis were observed in similar frequency in EOGBSS and LOGBSS. Leukopenia was more frequent in EOGBSS. Bulkowstein et al. (2016) [39] observed a significantly higher incidence of leukopenia and thrombocytopenia in early-onset sepsis and leukocytosis in late-onset sepsis. However, these are non-specific markers with low sensitivity and specificity in diagnosing sepsis [40, 41]. The level of support needed was compared between EOGBSS and LOGBSS. EOGBSS cases were more likely to need respiratory support (83% vs. 36%, *p* < 0.001). Other requirements, such as central lines, fluid boluses, inotropes, blood products, and mechanical ventilation, were similar between EOGBSS and LOGBSS.

The overall mortality was 8% (11% among preterm and 5% among term babies). The published studies have reported mortality rates of 2–3% and 1–3% among term babies with EOGBSS and LOGBSS, respectively [16, 19]. Premature babies have considerably higher mortality rates ranging from 20–30% [16, 19, 20]. Yeo et al. (2017) [19] observed that the mortality risk persisted beyond hospital discharge. Similarly, Madrid et al. reported an overall case fatality rate of 8%.

Survivors of GBSS are at risk of adverse neurodevelopmental outcomes. The risk is significantly higher among extremely premature babies and those with GBS meningitis [20, 42].

The files of all enrolled babies were reviewed for documented follow-ups beyond 18 months of age. For babies with at least three visits to a paediatrician after 18 months of age, documentation was reviewed for any evidence of major neurodisability, defined as cerebral palsy, major hearing/visual deficit, or intellectual disability as documented in the file. Overall, 18% of the babies were documented to have major neurodisability. There was no difference in major neurodisability (9.8% with EOGBSS, 13.5% with LOGBSS, *P* = 0.58) or combined death and major neurodisability (19% in EOGBSS vs. 15.8% in LOGBSS, *P* = 0.64) between the two types of sepsis. In a systematic review, Kohli-Lynch et al. (2017) [43] observed neurodevelopmental impairment among 18% of GBS meningitis survivors.

Meningitis (adjusted OR 11.53, *p* = 0.01), the need for fluid bolus (adjusted OR 16.8, *p* = 0.01), and peak CRP levels (adjusted OR 1.01, *p* = 0.03) emerged as significant predictors of combined mortality and neurodisability. These associations remained significant after adjusting for birth weight, gestational age, sex, leukopenia, thrombocytopenia, and the need for positive pressure ventilation at birth.

The latest Cochrane review [44] suggested that CRP alone may lack sufficient accuracy as a diagnostic tool for neonatal sepsis. However, a newer systematic review [45] found that CRP levels ≥ 60 mg/L show promising discriminatory ability for sepsis in neonates in low- and middle-income settings (AUC 0.87, 95% CI 0.76 to 0.91). The predictive value of CRP for sepsis-related mortality remains debated. While Deaneva et al. (2024) [46] identified CRP ≥ 5.75 mg/L as significantly predictive of mortality (OR = 15.56; 95% CI 2.59 to 93.57; *p* = 0.001), Liang et al. (2023) [47] found little correlation between CRP levels and sepsis mortality. Our finding of CRP as a predictor of neurodisability align with the work of Lee et al. (2021) [48] who observed that elevated inflammatory markers, including CRP, were associated with lower Bayley-III scores at 18 months corrected age in preterm infants with sepsis. Similarly, Kuban et al.(2017) [49] demonstrated that high CRP correlates to cognitive deficits at 10 years in extreme premature babies.

## Limitations

There are some inherent limitations to the retrospective design of this study that should be acknowledged. We did not have access to current data on maternal GBS colonisation rates, which could have provided important context for interpreting the results. Our study only included cases with GBS bacteraemia, and therefore, isolated cases of meningitis without bacteraemia may have been missed. Nanduri et al. (2019) [16] have reported that 0.1% of EOGBSS and 7.2% of LOGBSS present as meningitis without bacteremia. Although the missed EOGBSS cases would be negligible, the study may have missed 2–3 cases of LOGBSS during the whole 5-year period. Moreover, Qatar has a significant expatriate population, which increases the possibility of missing cases who travelled outside Qatar before three months of age. Therefore, the incidence of LOGBSS was slightly underestimated. Additionally, relying solely on physician documentation to assess neurodevelopmental outcomes might miss milder cases, leading to underestimation. Despite these limitations, our study provides important insights into the epidemiology and outcomes of GBSS in Qatar.

## Conclusions

This 5-year, multi-center retrospective study provides updated, population-representative estimates of EOGBSS (0.58/1000 LB) and LOGBSS (0.33/1000 LB) in Qatar, based on over 90% of births between 2015 and 2020. Our findings show no significant change in EOGBSS incidence since 2003, and mortality remains substantial at 8%, with at least 11% of survivors facing adverse neurodevelopmental outcomes. These results add valuable insights to the limited data on regional sepsis rates and outcomes, emphasizing the need for a national prospective surveillance system to support perinatal healthcare improvements. Further studies should explore targeted prevention strategies and long-term neurodevelopmental outcomes.

## Data Availability

The datasets supporting the conclusions of this article are available in the ‘Open Science Framework’ via https://osf.io/agtvr/?view_only=01383dcb35a14196b315ff65564bd79d.

## Abbreviations

WWRC: Women's Wellness and Research Centre
AWH: Al Wakra Hospital
AKH: Al Khor Hospital
TCH: The Cuban Hospital
GBS: Group B Streptococcus
EOGBSS: Early-onset GBS sepsis
LOGBSS: Late-onset GBS sepsis
LB: Live Birth
CRP: C-Reactive Protein
CSF: Cerebrospinal Fluid
LP: Lumbar Puncture
SD: Standard Deviation
MSAF: Meconium-Stained Amniotic Fluid
NICU: Neonatal Intensive Care Unit
SROM: Spontaneous rupture of membranes
PPV: Positive Pressure Ventilation
WBC: White Blood Cells
